# Prevalence, antimicrobial resistance, and genomic characterization of non-typhoidal *Salmonella* in Thai native Blackbone chickens

**DOI:** 10.14202/vetworld.2026.493-510

**Published:** 2026-02-10

**Authors:** Kanit Assawatheptawee, Anong Kiddee, Uttapoln Tansawai, Taradon Luangtongkum, Pannika R. Niumsup

**Affiliations:** 1 Department of Microbiology and Parasitology, Faculty of Medical Science, Naresuan University, Phitsanulok, 65000, Thailand; 2 Division of Microbiology and Parasitology, School of Medical Sciences, University of Phayao, Phayao, 56000, Thailand; 3 Department of Science, Faculty of Science and Agricultural Technology, Rajamangala University of Technology Lanna Phitsanulok, Phitsanulok, 65000, Thailand; 4 Department of Veterinary Public Health, Faculty of Veterinary Science, Chulalongkorn University, Bangkok, 10330, Thailand; 5 Center of Excellence in Medical Biotechnology, Faculty of Medical Science, Naresuan University, Phitsanulok, 65000, Thailand

**Keywords:** antimicrobial resistance, Blackbone chicken, food safety, genomic characterization, native poultry, non-typhoidal *Salmonella*, poultry meat, prevalence, *Salmonella* Bovismorbificans, *Salmonella* Weltevreden, Thailand, whole-genome sequencing, zoonotic pathogens

## Abstract

**Background and Aim:**

Native Blackbone chickens (*Gallus gallus domesticus*) are increasingly consumed in Southeast Asia because of their perceived health benefits. However, information on the epidemiology, antimicrobial resistance (AMR), and genomic characteristics of non-typhoidal *Salmonella* associated with this traditional poultry system remains limited. This study aimed to determine the prevalence, serovar distribution, antimicrobial susceptibility patterns, and genomic features of *Salmonella* isolated from feces and meat of Thai native Blackbone chickens.

**Materials and Methods:**

A prospective longitudinal study was conducted between August 2020 and July 2021 on a small-scale Blackbone chicken farm in Phitsanulok province, Thailand. A total of 2,258 samples, comprising 1,755 fecal and 503 meat samples, were collected from chickens aged 1–50 weeks. *Salmonella* isolation was performed using standard culture and biochemical methods, with confirmation by *invA* using polymerase chain reaction. Serovars were determined by slide agglutination. Antimicrobial susceptibility was assessed against 14 antimicrobial agents using disk diffusion and broth microdilution methods. Six representative isolates were subjected to whole-genome sequencing (WGS) for multilocus sequence typing, detection of AMR and virulence genes, plasmid analysis, and phylogenetic comparison.

**Results:**

The overall prevalence of *Salmonella* was 6.3% (142/2,258), with detection rates of 4.7% in fecal samples and 11.9% in meat samples. *Salmonella* Bovismorbificans was the predominant serovar (64.1%), followed by *S*. Corvallis (20.4%) and *S*. Weltevreden (9.2%). More than half of the isolates (52.8%) exhibited resistance to at least one antimicrobial, most commonly streptomycin (46.5%), while resistance to critically important antimicrobials was not observed. Multidrug resistance (MDR) was rare (1.4%). WGS revealed that *S*. Bovismorbificans isolates belonged to sequence type ST1499, whereas *S*. Weltevreden was identified as ST365. Despite limited AMR, all sequenced isolates carried numerous virulence-associated genes linked to adhesion, invasion, and intracellular survival.

**Conclusion:**

Thai native Blackbone chickens harbor non-typhoidal *Salmonella*, including emerging and potentially invasive lineages, with low levels of MDR but substantial virulence potential. These findings highlight the zoonotic risk posed by traditional poultry systems and underscore the importance of continued surveillance and genomic monitoring to support food safety and One Health strategies.

## INTRODUCTION

Non-typhoidal *Salmonella* spp. are Gram-negative bacteria capable of colonizing or infecting a wide range of animals and humans. They represent major zoonotic pathogens of global public health significance. Food-producing animals, particularly poultry, swine, and cattle, serve as important reservoirs, facilitating transmission to humans through direct contact or by consuming contaminated food or water. In addition, slaughterhouse and processing environments are recognized as critical points for *Salmonella* contamination of animal-derived food products [1]. Molecular epidemiological studies from several countries, including Thailand, have demonstrated close genetic relatedness among *Salmonella* isolates from humans, poultry meat, and environmental sources, supporting the occurrence of inter-source transmission [2–4].

Human salmonellosis is commonly characterized by mild, self-limiting gastroenteritis with low mortality and often does not require antimicrobial treatment. However, untreated infections may progress to invasive disease, including bacteremia, meningitis, and other extraintestinal manifestations, which can be fatal. Severe salmonellosis has also been associated with an increased risk of colon cancer [5]. Children, elderly individuals, and immunocompromised patients are particularly vulnerable to invasive *Salmonella* infections [1]. According to the World Health Organization (WHO), *Salmonella* spp. rank fourth among the top 10 causes of foodborne illness in Southeast Asia, accounting for more than 15 million cases and approximately 16,000 deaths annually [6]. Reflecting this burden, the WHO has classified *Salmonella* spp. as a “high-priority” pathogen for antibiotic research and public health interventions [7].

Although human salmonellosis is a worldwide disease, severe effects from *Salmonella* spp. infections most frequently occur in low- and middle-income countries, such as those in Africa, South Asia and South-East Asia. International tourists who travel to these regions may be infected with *Salmonella* spp. The incidence of *Salmonella* gastroenteritis has been reported in children and adults throughout the year in different parts of Thailand. However, these may be underestimated because symptoms are usually self-limited and may not be formally diagnosed. Invasive *Salmonella* spp. infections have also been reported in all regions of Thailand [3, 8–10].

The global rise of antimicrobial-resistant *Salmonella* spp., including multidrug-resistant (MDR) strains, represents a growing threat to human and animal health. Infections caused by MDR *Salmonella* are associated with prolonged illness, therapeutic failure, increased healthcare costs, and higher mortality rates [1]. In Thailand, surveillance studies have documented substantial levels of resistance among *Salmonella* isolates from food animals. Reports indicate that 12.2%–41.2% of samples from broilers, pigs, ducks and chicken meat were *Salmonella*-positive, with MDR rates ranging from 23.2% to 30.4% [11–13]. Even higher MDR prevalence (52.8%–77.5%) has been reported among clinical isolates from Thai patients [9, 10], with notable resistance to ciprofloxacin and third-generation cephalosporins—antimicrobials considered critically important for human medicine [8–10].

Native chickens are widely raised in underdeveloped and developing countries and play an important role in supporting household livelihoods. In Thailand, diverse native chicken breeds exist, including Blackbone chickens, which are distinguished by their white feathers and black beak, bones, and skin, and their black meat. Consumption of Blackbone chickens has increased due to their perceived health benefits, including antioxidant properties, low cholesterol content, and potential neuroprotective effects demonstrated in experimental models [14–16].

Several pathogenic bacteria, including *Salmonella* spp., naturally colonize the intestinal tracts of native chickens raised in free-range or backyard systems. Although birds often remain asymptomatic, they can shed pathogens into the environment, posing risks to humans and other animals. *Salmonella* has been reported in native chickens from multiple countries, including the Philippines, Malaysia, Iran, and China [17–20]. Backyard poultry have also been implicated in salmonellosis outbreaks, and isolates from these systems are often genetically similar to strains causing human disease, highlighting their zoonotic potential [21–23].

Despite extensive research on *Salmonella* in commercial poultry systems, data from small-scale and native poultry farms in Thailand remain scarce. Earlier studies reported a low prevalence (0.67%) of *Salmonella* in Thai native chickens [24], while our previous work identified a 5.4% prevalence in a small-scale duck farm [25]. These findings underscore the need for comprehensive investigations into *Salmonella* epidemiology, antimicrobial resistance (AMR), and zoonotic risk within traditional poultry production systems.

Despite the well-documented role of commercial poultry systems in the epidemiology of non-typhoidal *Salmonella*, traditional and native poultry production systems remain markedly under-investigated, particularly in Southeast Asia. Existing studies from Thailand have largely focused on cross-sectional sampling of commercial broilers, slaughterhouses, or clinical isolates, providing limited insight into *Salmonella* dynamics within small-scale or native chicken farms. Where native poultry have been examined, investigations were often restricted to single time points, small sample sizes, or basic phenotypic characterization, without integrating longitudinal surveillance or genomic analysis. Consequently, critical questions remain unanswered regarding age-related shedding patterns, contamination of meat during production, and the persistence of specific *Salmonella* serovars throughout the rearing cycle in native chicken systems.

Moreover, information on the AMR and virulence potential of *Salmonella* circulating in Blackbone chickens is particularly scarce. Blackbone chickens are increasingly consumed for perceived health-promoting properties, yet their microbiological safety has received little scientific attention. The absence of genomic data from *Salmonella* isolates originating from these birds limits understanding of their evolutionary relationships to strains infecting humans, animals, and the environment. Without whole-genome sequencing (WGS) data, it is not possible to determine whether native poultry harbor emerging or invasive lineages, silently carry resistance determinants, or serve as reservoirs of virulence genes with zoonotic relevance. This lack of integrated phenotypic and genomic evidence represents a significant gap in national surveillance efforts and hinders risk assessment under a One Health framework.

The present study was designed to address these knowledge gaps by comprehensively characterizing non-typhoidal *Salmonella* in Thai native Blackbone chickens raised under small-scale production conditions. Specifically, this study aimed to (i) determine the prevalence and age-associated shedding patterns of *Salmonella* spp. in fecal samples throughout the rearing period, (ii) assess the occurrence of *Salmonella* contamination in meat samples at different production stages, and (iii) identify the circulating *Salmonella* serovars in this native poultry system. In addition, the study sought to (iv) evaluate antimicrobial susceptibility profiles and the occurrence of MDR among isolates and (v) elucidate genomic characteristics, including sequence types, AMR genes (ARGs), virulence determinants, plasmid content, and phylogenetic relationships using WGS.

By integrating longitudinal sampling with phenotypic and genomic analyses, this study aimed to generate baseline molecular epidemiological data on *Salmonella* in Blackbone chickens. The findings are intended to inform food safety risk assessments, strengthen AMR surveillance, and improve understanding of the zoonotic potential of native poultry systems in Thailand and comparable settings.

## MATERIALS AND METHODS

### Ethical approval

This study was conducted in full compliance with institutional, national, and international guidelines governing the ethical use of animals in research. The study protocol was reviewed and approved by the Animal Care and Use Committee of Chulalongkorn University under animal use protocol number 2031019 (approval date: July 22, 2020). All experimental and sampling procedures adhered strictly to the principles of animal welfare, including the minimization of stress, discomfort, and disturbance to animals during routine farm activities and sample collection.

Fecal sampling was non-invasive and involved the collection of freshly voided feces from the ground, thereby avoiding direct handling or restraint of birds. Meat samples were obtained from birds slaughtered as part of standard farm management and commercial processing practices, and no animals were euthanized specifically for research purposes. Prior to commencement of the study, verbal informed consent was obtained from the farm owner for on-farm observations, interviews, and sample collection.

All laboratory procedures involving *Salmonella* spp. were performed in a Biosafety Level 2 laboratory in accordance with institutional biosafety policies and national regulations on the handling of pathogenic microorganisms. Personnel involved in laboratory work were trained in biosafety and biosecurity procedures, and appropriate personal protective equipment was used at all times. Decontamination of biological waste and materials was carried out using approved methods, including autoclaving, to prevent environmental contamination and occupational exposure.

The study did not involve human participants, human biological samples, or the collection of personal or identifiable data. Therefore, approval from a human research ethics committee was not required. Overall, the study was designed and implemented to ensure ethical integrity, biosafety, and responsible conduct of research, consistent with the principles of the One Health framework.

### Study period and location

The study was carried out over a 12-month period from August 2020 to July 2021 at a Blackbone chicken farm located in Phitsanulok province (16°49′N, 100°15′E), approximately 377 km north of Bangkok in the lower northern region of Thailand. The area has a tropical climate characterized by a hot, dry season from November to April and a hot, wet season from May to October. Mean temperatures range from 27.1°C during the dry season to 28.6°C during the wet season, with an average annual humidity of 72% and an annual rainfall of approximately 1662 mm, predominantly occurring during the wet season.

### Study design and farm management

A prospective longitudinal surveillance study was conducted to evaluate the prevalence and antimicrobial resistance of *Salmonella* spp. in Thai native Blackbone chickens from a single small-scale farm that has been in operation since 2015. All chickens originated from the same hatchery and were reared in an open-house system enclosed with netting. Rice straw was used as bedding material, and birds were managed under standardized husbandry conditions. Commercial feed and filtered groundwater were provided ad libitum throughout the rearing period. Biosecurity measures were limited, with disposable shoe covers being the only precaution applied before farm entry. Tylosin was administered infrequently, for short durations, and only when birds showed clinical signs of illness. During the sampling period, approximately 1% of the flock showed unexplained mortality.

### Sample size calculation and sampling scheme

Sample size determination was performed using the Epitools online software (http://epitools.ausvet. com.au). As the true prevalence of *Salmonella* spp. in Blackbone chickens was unknown, an expected prevalence of 50% was assumed, with a 95% confidence interval (CI) and a 10% margin of error. This calculation indicated a minimum requirement of 97 fecal samples per sampling time point. Accordingly, fresh fecal samples were randomly collected from approximately 10% of the flock (about 122–155 birds) from a population of approximately 1,500 chickens.

### Fecal and meat sample collection

Fresh fecal samples were collected weekly from the ground from chickens aged 1–5 weeks using sterile spoons and placed into disposable containers. Subsequent sampling was conducted at 7, 10, 14, 16, 22, 28, 40, and 50 weeks of age. Care was taken to avoid feces that had come into contact with the ground surface.

Meat samples were collected from all birds slaughtered at 7, 10, and 16 weeks of age. A comparable number of meat samples was randomly collected when the remaining birds were slaughtered at the end of the production cycle (50 weeks of age). Chicken carcasses were individually placed in sterile plastic bags, transported on ice, and processed within 24 h of collection.

### Isolation and culture of *Salmonella* spp.

For fecal samples, 1 g of each sample was enriched in 9 mL buffered peptone water (BPW) (Difco™, MD, USA) and incubated at 37°C for 24 h. For meat samples, 25 g of tissue was placed in a sterile bag containing 225 mL BPW, homogenized for 2 min using a stomacher, and incubated at 37°C for 24 h. Subsequently, 0.1 mL of enriched BPW was transferred into 9.9 mL Rappaport–Vassilliadis soya peptone (RVS) broth and incubated at 42°C ± 1°C for 24 h, while 1 mL of BPW was transferred into 9 mL tetrathionate (TT) broth (Oxoid, Hampshire, UK) and incubated at 37°C ± 1°C for 24 h. A loopful from each incubated broth was streaked onto Xylose Lysine Deoxycholate (XLD) agar (Oxoid) and incubated overnight at 37°C. *Salmonella* Typhimurium DMST562, obtained from the Department of Medical Sciences, Ministry of Public Health, Bangkok, Thailand, was used as a positive control strain.

### Biochemical and molecular confirmation of *Salmonella* spp.

Presumptive *Salmonella* colonies on XLD agar appeared as red colonies with black centers. One suspect colony per sample was identified biochemically using API 20E (bioMérieux SA, Marcy-l’Étoile, France) and subsequently confirmed by polymerase chain reaction (PCR) amplification of the *invA* gene (284 bp) using primers invA-F (5′-GTGAAATTATCGCCACGTTCGGGCAA-3′) and invA-R (5′-TCATCGCACCGTCAAAGGAACC-3′), as previously described [26]. A single colony was suspended in 300 µL sterile water and used as the PCR template. Amplification was performed in a total volume of 20 µL containing 1 µL template DNA, 1× PCR buffer, 1.5 mM MgCl^2^, 0.2 mM dNTPs, 0.5 µM of each primer, and 1 U *Taq* polymerase (Vivantis Technologies, Selangor, Malaysia). The PCR cycling conditions consisted of an initial denaturation at 95°C for 2 min, followed by 30 cycles of denaturation at 95°C for 30 s, annealing at 45°C for 1 min, extension at 72°C for 45 s, and a final extension at 72°C for 7 min. PCR products were analyzed by electrophoresis on 2% agarose gels.

All confirmed *Salmonella* isolates were preserved at −80°C in VIABANK™ Bacterial Storage Beads (Medical Wire & Equipment Co. Ltd., Wiltshire, England) for further analyses.

### Serovar identification

Initial serogrouping of isolates was performed by slide agglutination using *Salmonella* polyvalent O antiserum (A–I), somatic antigens (OMA, OMB, and OMC), and serogroup-specific antisera (A, B, C, D, E, and F) (S & A Reagents Lab, Bangkok, Thailand). All *Salmonella* spp. isolates (n = 142) were subsequently submitted to the Center for Antimicrobial Resistance Monitoring in Foodborne Pathogens (in cooperation with WHO), Faculty of Veterinary Science, Chulalongkorn University, Thailand, for definitive serovar identification.

### Antimicrobial susceptibility testing

Antimicrobial susceptibility testing was performed using the disk diffusion method in accordance with Clinical and Laboratory Standards Institute (CLSI) guidelines [27]. Isolates were grown overnight on Trypticase soy agar at 37°C. Three to five colonies were adjusted to a 0.5 McFarland standard (1.5 × 10^8^ CFU/mL) in normal saline and evenly spread onto Mueller–Hinton agar (MHA) plates (4 mm thickness, pH 7.2–7.4; Oxoid). Antibiotic disks were applied, and plates were incubated at 35 ± 2°C for 16–18 h. Inhibition zone diameters were measured and interpreted according to CLSI criteria (CLSI M100, 30th ed., 2020) [27].

The antimicrobial agents tested included β-lactams (ampicillin 10 µg, cefazolin 30 µg, cefuroxime 30 µg, cefotaxime 30 µg, and ceftazidime 30 µg), aminoglycosides (streptomycin 10 µg and gentamicin 10 µg), tetracycline (doxycycline 30 µg), quinolone (ciprofloxacin 5 µg), phenicol (chloramphenicol 30 µg), folate pathway antagonist (trimethoprim/sulfamethoxazole 25 µg), fosfomycin 200 µg, and nitrofurantoin 300 µg (Oxoid). All disks were stored at 2°C–4°C in sealed containers protected from light according to the manufacturer’s instructions. *Escherichia coli* ATCC25922 was used as the quality control strain. Zone diameter interpretive criteria are provided in Table S1. MDR was defined as resistance to three or more antimicrobial classes [28].

### Colistin minimum inhibitory concentration (MIC) determination

Colistin susceptibility was determined by broth microdilution to establish the MIC following CLSI guidelines [27]. Twofold serial dilutions of colistin (Sigma-Aldrich, MO, USA) were prepared in 200 µL cation-adjusted Mueller–Hinton broth (Oxoid) in microtiter plates. Bacterial suspensions were added to achieve a final inoculum of 5 × 10^5^ CFU/mL per well, and plates were incubated at 35 ± 2°C for 18–20 h. The MIC was defined as the lowest concentration inhibiting visible growth. Isolates with MIC ≥ 4 µg/mL were classified as colistin resistant (Table S2) (CLSI M100, 30th ed., 2020) [27].

### Genomic DNA extraction and WGS

Six *Salmonella* spp. isolates were selected for WGS based on antimicrobial resistance phenotypes and sample origins. Isolates were cultured overnight in Tryptic Soy Broth at 37°C. Genomic DNA was extracted using the TIANamp Genomic DNA Kit (Tiangen®, Beijing, China) according to the manufacturer’s instructions. DNA purity was assessed by A260/280 ratios (≥1.8), and DNA concentration was quantified using a NanoDrop spectrophotometer (Thermo Fisher Scientific, MA, USA). DNA concentrations ranged from 18.3 to 25.4 ng/µL (Table S3). Purified DNA samples were submitted to a commercial facility (Macrogen, Seoul, Korea) for library preparation using the Nextera XT DNA Library Preparation Kit and sequenced on the Illumina NovaSeq platform with 150 bp paired-end reads (Illumina Inc., San Diego, USA).

### Genome assembly, annotation, and in silico analyses

All bioinformatic analyses were conducted using default parameters unless otherwise specified. Raw sequencing reads were assembled using Unicycler v0.4.8 [29] via the Bacterial and Viral Bioinformatics Resource Center (https://www.bv-brc.org). Genome annotation was performed using Prokka v1.14.6 [30]. Species identification was confirmed by average nucleotide identity analysis based on ANIb using the JSpeciesWS online server [31]. Multilocus sequence typing was conducted using MLST v2.0.9 [32], and antimicrobial resistance genes were identified using ResFinder v4.6.0 (identity ≥90%, coverage ≥80%) [33] through the Center for Genomic Epidemiology (https://www.genomicepidemiology.org). MobileElementFinder v1.0.3 was used to identify plasmid replicon types and the presence of the colicin Ib gene (cib) [34]. Virulence-associated genes were identified using the virulence factor analyzer based on the Virulence Factor Database 2022 (VFDB) with default settings [35].

### Phylogenetic analysis based on core genome SNPs

Phylogenetic relationships among *Salmonella* spp. isolates were assessed using core genome single-nucleotide polymorphism analysis. Assembled genomes of *Salmonella* Bovismorbificans (n = 5) and *Salmonella* Weltevreden (n = 1) from this study were compared with publicly available *Salmonella* genomes retrieved from the NCBI database. Core genes were identified using Roary v3.13.0 [36] based on Prokka-annotated GFF3 files. Concatenated core gene alignments were used to construct a maximum-likelihood phylogenetic tree with CSIPhylogeny v1.4 [37], and the resulting tree was visualized using Interactive Tree of Life (iTOL v7) [38].

### Statistical analysis

Descriptive statistics were used to summarize prevalence and AMR data. Differences in detection rates between fecal and meat samples and among sampling periods were analyzed using chi-square or Fisher’s exact tests. Statistical analyses were performed using SPSS version 17.0, with p < 0.05 considered statistically significant.

## RESULTS

### Prevalence of *Salmonella* spp. in Blackbone chickens

**Figure 1:**
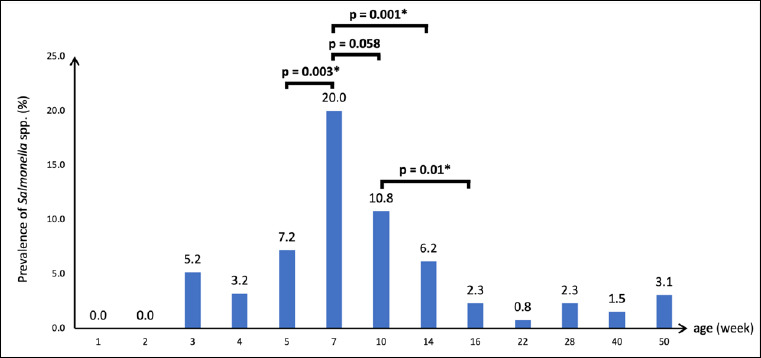
*Salmonella* spp. prevalence in fecal samples of Blackbone chickens.

The overall prevalence of *Salmonella* spp. in Blackbone chickens was 6.3% (95% CI: 5.3%–7.4%; 142/2,258). At the farm level, *Salmonella* spp. were detected in 4.7% (82/1,755) of fecal samples (Table 1). No *Salmonella* was detected in feces from chickens during the first 1–2 weeks of age. Fecal shedding was first observed in 3-week-old birds (5.2%) and increased significantly at 7 weeks of age, reaching the highest prevalence of 20.0% (*p* < 0.05) (Figure 1). Thereafter, shedding declined to 10.8% at 10 weeks and decreased significantly at 14 weeks (6.2%, p < 0.05). This downward trend continued until the end of the rearing period, with a detection rate of 3.1% at 50 weeks of age.

**Table 1:** *Salmonella* spp. prevalence and serovar distribution in fecal and meat samples of Blackbone chickens.

Age (week)	Feces			Meat		
	
	No.	*Salmonella* spp.	Serovars	No.	*Salmonella* spp.	Serovars
1	155	0	–	–	–	–
2	155	0	–	–	–	–
3	155	8 (5.2) (2.3–9.9)	Bovismorbificans (n = 7) Weltevreden (n = 1)	–	–	–
4	125	4 (3.2) (0.9–8.0)	Bovismorbificans (n = 4)	–	–	–
5	125	9 (7.2) (3.3–13.2)	Bovismorbificans (n = 6) Weltevreden (n = 2) Corvallis (n = 1)	–	–	–
7	130	26 (20.0) (13.5–27.9)	Bovismorbificans (n = 15) Corvallis (n = 6) Weltevreden (n = 2) Stanley (n = 2) Typhimurium (n = 1)	125	27 (21.6) (14.7–29.8)	Bovismorbificans (n = 20) Weltevreden (n = 5) Stanley (n = 2)
10	130	14 (10.8) (6.0–17.4)	Corvallis rats (n = 13) Weltevreden (n = 1)	126	11 (8.7) (4.4–15.1)	Bovismorbificans (n = 9) Weltevreden (n = 1) Stanley (n = 1)
14	130	8 (6.2) (2.7–11.8)	Bovismorbificans (n = 8)	–	–	–
16	130	3 (2.3) (0.5–6.6)	Bovismorbificans (n = 3)	126	20 (15.9) (10.0–23.4)	Bovismorbificans (n = 17) Kentucky (n = 2) Corvallis (n = 1)
22	130	1 (0.8) (0.1–4.2)	Corvallis (n = 1)	–	–	–
28	130	3 (2.3) (0.5–6.6)	Corvallis (n = 3)	–	–	–
40	130	2 (1.5) (0.2–5.4)	Corvallis (n = 1) Serogroup C (n = 1)	–	–	–
50	130	4 (3.1) (0.8–7.7)	Corvallis (n = 3) Weltevreden (n = 1)	126	2 (1.6) (0.2–5.6)	Bovismorbificans (n = 2)
Total	1,755	82 (4.7) (3.1–5.8)	Bovismorbificans (n = 43) Corvallis (n = 28) Weltevreden (n = 7) Stanley (n = 2) Typhimurium (n = 1) Serogroup C (n = 1)^a^	503	60 (11.9) (9.2–15.1)	Bovismorbificans (n = 48) Weltevreden (n = 6) Stanley test (n = 3) Kentucky (n = 2) Corvallis (n = 1)

a Serovar identification was untypeable; however, typing performed by agglutination with *Salmonella* antiserum revealed that this isolate belonged to serogroup C. CI = Confidence interval.

**Figure 2:**
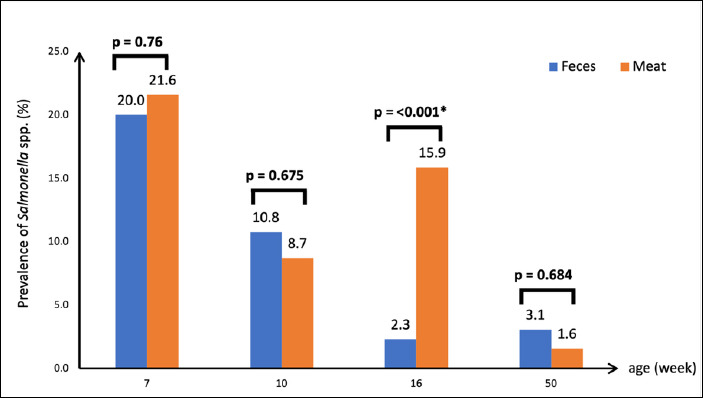
*Salmonella* spp. prevalence in fecal and meat samples of Blackbone chickens.

In meat samples, *Salmonella* spp. were detected in 11.9% (60/503) of samples collected from chickens of all age groups, although detection rates varied (Table 1). No significant differences were observed between fecal and meat samples within the same age groups. However, at 16 weeks of age, the prevalence of *Salmonella* in meat samples was significantly higher than that in fecal samples (15.9% vs 2.3%, p < 0.001) (Figure 2).

### Serovar distribution of *Salmonella* spp.

Serovar analysis identified six distinct *Salmonella* serovars among 141 isolates, while one isolate belonged to serogroup C (Tables 1 and 2). Similar serovar distributions were observed in fecal and meat samples. *Salmonella* Bovismorbificans was the predominant serovar, accounting for 64.1% (91/142) of all isolates, followed by *S*. Corvallis (20.4%, 29/142) and *S*. Weltevreden (9.2%, 13/142). Minor serovars included *Salmonella* Stanley, *Salmonella* Kentucky, and *S*. Typhimurium, each represented by one to five isolates.

**Table 2:** Antibiotic resistance profiles of *Salmonella* spp. isolated from Blackbone chickens (n = 142).

Antibiotic resistance profile	Number of antibiotic classes	Number of isolates, n (%) (95% CI)	Serovar (n)
1. KZ-S-DO-F	4	1 (0.7) (0.02–3.9)	Bovismorbificans (n = 1)^a^
2. AMP-KZ-S-CN	3	1 (0.7) (0.02–3.9)	Bovismorbificans (n = 1)^a^
3. AMP-KZ-S	2	3 (2.1) (0.4–6.0)	Corvallis (n = 3)
4. AMP-KZ-DO	2	4 (2.8) (0.8–7.1)	Bovismorbificans (n = 3), Weltevreden (n = 1)
5. AMP-KZ	1	3 (2.1) (0.4–6.0)	Corvallis (n = 2), Weltevreden (n = 1)
6. AMP-S	2	1 (0.7) (0.02–3.9)	Corvallis (n = 1)
7. KZ	1	1 (0.7) (0.02–3.9)	Bovismorbificans (n = 1)
8. S	1	60 (42.3) (34.0–50.8)	Bovismorbificans (n = 39), Corvallis (n = 18), Stanley (n = 1), Typhimurium (n = 1), Serogroup C (n = 1)
9. TMP/SMX	1	1 (0.7) (0.02–3.9)	Stanley (n = 1)
Subtotal (resistant isolates)		75 (52.8) (44.3–61.2)	
Susceptible to 13 antibiotics tested		67 (47.2) (38.8–55.7)	
Total		142	

AMP = Ampicillin, CN = Gentamicin, DO = Doxycycline, F = Nitrofurantoin, KZ = Cefazolin, S = Streptomycin, TMP/SMX = Trimethoprim/ sulfamethoxazole.

a Isolates were defined as multidrug-resistant.

### Antimicrobial susceptibility patterns

**Figure 3:**
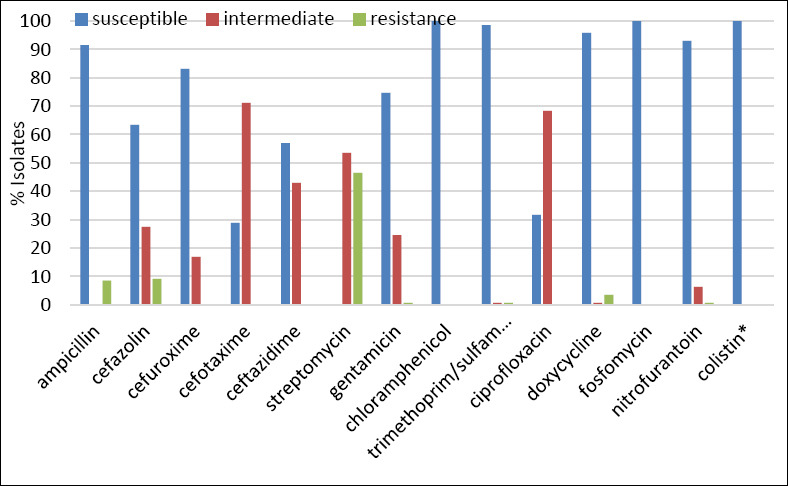
*Salmonella* spp. susceptibility test from Blackbone chickens (n = 142). *Susceptibility to colistin was determined using the broth microdilution method.

All *Salmonella* isolates were tested against 14 antimicrobial agents. Overall, 52.8% (75/142) of isolates exhibited resistance to at least one antibiotic (Table 2). The highest resistance rate was observed for streptomycin (46.5%), followed by cefazolin (9.1%) and ampicillin (8.5%) (Figure 3). Resistance to gentamicin, trimethoprim/ sulfamethoxazole, doxycycline, and nitrofurantoin was detected at low frequencies (0.7%–3.5%). All isolates were fully susceptible to cefuroxime, cefotaxime, ceftazidime, chloramphenicol, ciprofloxacin, and fosfomycin.

Nine distinct resistance profiles were identified, with most resistant isolates (42.3%, 60/142) displaying resistance to streptomycin alone (Table 2). MDR was rare, occurring in only 1.4% (n = 2) of *S*. Bovismorbificans isolates. All isolates were susceptible to colistin, with MICs ranging from 0.25 to 2 µg/mL (Figure 3; Table S2).

### WGS and genomic features

Six *Salmonella* isolates (four from feces and two from meat) were selected for WGS based on resistance phenotypes and sample origins. High-quality sequencing data were obtained, with average Q20 and Q30 scores of 95.9% and 89.7%, respectively (Table S3). Genome assemblies averaged 4.85 Mb in size, with a mean of 53 contigs, an average sequencing depth of 283×, an N50 of 361,987 bp, and a GC content of approximately 52.1%.

**Figure 4:**
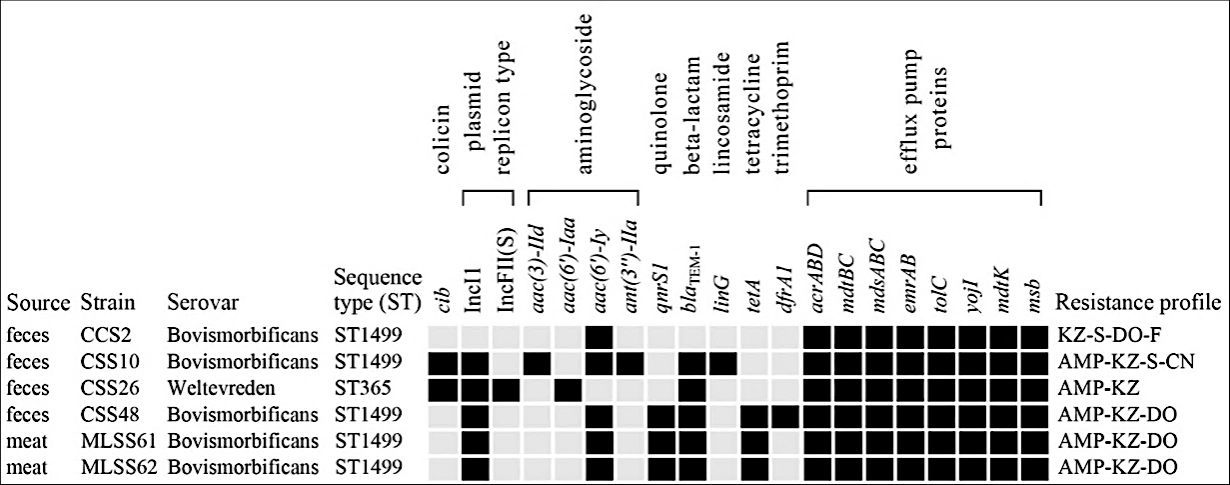
Sequence types**,** antibiotic-resistant genes, plasmid replicon types, and colicin Ib (*cib*) in Blackbone chicken isolates of selected *Salmonella* spp. (n = 6). The present and absent genes are shown in black and gray, respectively. AMP = Ampicillin, KZ = Cefazolin, S, streptomycin, CN = Gentamicin, DO = Doxycycline, F = Nitrofurantoin.

Average nucleotide identity analysis confirmed all isolates as *Salmonella* *enterica* (99.9% identity). In silico MLST identified all *S*. Bovismorbificans isolates as sequence type ST1499, whereas the single *S*. Weltevreden isolate (CSS26) belonged to ST365 (Figure 4).

### ARGs and plasmid profiles

Genomic analysis revealed ARGs associated with six antibiotic classes (Figure 4). All isolates harbored aminoglycoside resistance genes, including *aac(6′)-Iy*, *aac(6′)-Iaa*, *aac(3)-IId*, or *ant(3′′)-IIa*, with *aac(6′)-Iy* being the most prevalent (83.3%). The β-lactamase gene *bla*_TEM-1_ was detected in five isolates. Three isolates additionally carried *qnrS1* and *tet(A)*, conferring reduced susceptibility to quinolones and tetracyclines, respectively. Genes conferring resistance to trimethoprim (*dfrA1*) and lincosamides (*linG*) were each identified in one isolate. All genomes also encoded multiple efflux pump systems.

Plasmid analysis showed that five isolates carried an IncI1 plasmid. Two of these isolates (one *S*. Bovismorbificans and one *S*. Weltevreden) harbored the *cib* gene encoding colicin Ib. The *S*. Weltevreden CSS26 isolate additionally carried an IncFII(S) plasmid, which exhibited 99% sequence identity to plasmid pSH17G0407 from *S*. Weltevreden ST365 isolated in China.

### Virulence gene profiles

**Figure 5:**
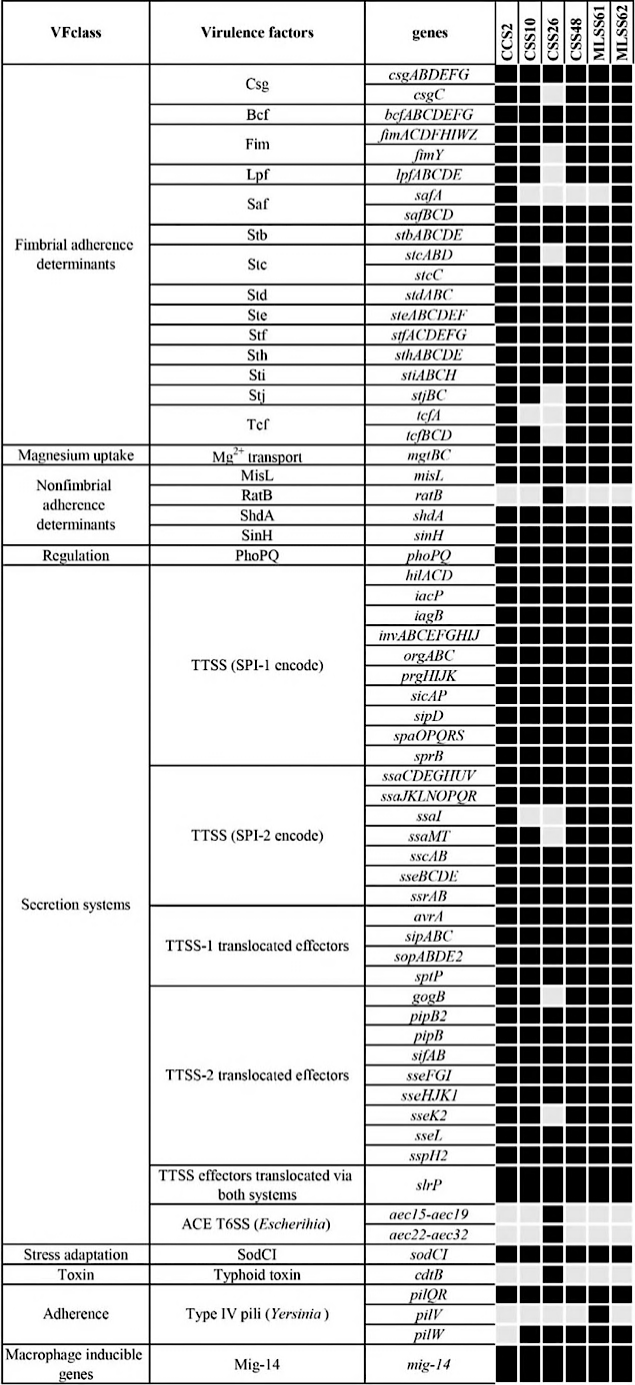
Virulence factor-associated genes in Blackbone chicken isolates of selected *Salmonella* spp. (n = 6). The present and absent genes are shown in black and gray, respectively.

Virulence factor analysis identified between 158 and 162 virulence-associated genes per isolate (Figure 5), with 79.6% of genes conserved across all strains. *S*. Bovismorbificans isolates from meat samples (MLSS61 and MLSS62) carried the highest number of virulence genes. All isolates possessed genes associated with adherence, invasion, intracellular survival, stress adaptation, and regulation. Core virulence determinants included fimbrial and non-fimbrial adhesins, type IV pili, macrophage survival genes, and regulators such as *phoPQ*. Type III secretion system genes encoded on *Salmonella* pathogenicity islands SPI-1 and SPI-2, along with their effector proteins, were highly conserved.

Notably, *S*. Weltevreden CSS26 lacked several fimbrial operons but uniquely possessed *ratB*, *cdtB*, and *aec* operons, which are associated with enhanced invasiveness and host cell damage.

### Phylogenetic relationships

**Figure 6:**
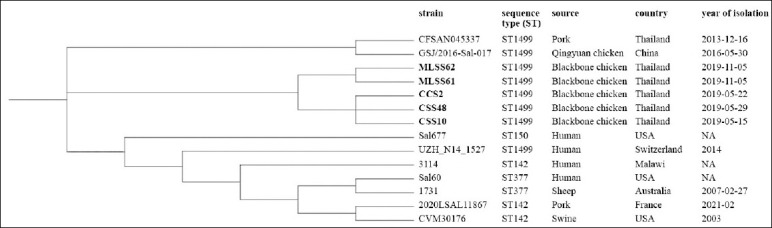
Core genome single-nucleotide polymorphism-based phylogenetic tree of *Salmonella* Bovismorbificans (CCS2, CSS10, CSS48, MLSS61, and MLSS62) from Blackbone chickens and additional genomes, which are publicly available in the National Center for Biotechnology Information database (Table S4).

Core genome single-nucleotide polymorphism(cgSNP)-based phylogenetic analysis revealed that *S*. Bovismorbificans isolates clustered into three distinct groups (Figure 6). All five isolates from Blackbone chickens formed a single cluster (Group II), distinct from isolates obtained from pork in Thailand and chickens in China (Group I), and from strains originating in Australia, Europe, Africa, and the USA (Group III).

**Figure 7:**
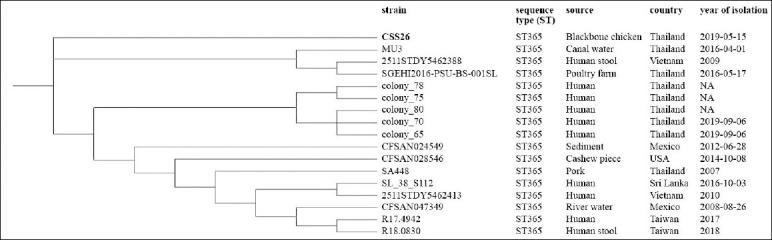
Core genome single-nucleotide polymorphism-based phylogenetic tree of *Salmonella* Weltevreden (CSS26) from Blackbone chickens and additional genomes, which are publicly available from the NCBI database (Table S5).

Phylogenetic analysis of *S*. Weltevreden isolates identified three major clusters based on geographic origin and source (Figure 7). The CSS26 isolate occurred as a singleton, separate from other ST365 strains recovered from humans, food, and environmental sources, indicating a genetically distinct lineage associated with Blackbone chickens.

## DISCUSSION

### Prevalence of *Salmonella* spp. in Blackbone chickens

*Salmonella* spp. can be isolated from various food-producing animals at various stages of production. This study investigated the presence of *Salmonella* spp. in native Thai Blackbone chickens. A previous study in China reported a 38.9% prevalence of *Salmonella* spp. in Blackbone chickens [39]. Our study revealed that *Salmonella* spp. had an overall prevalence of 6.3% in Blackbone chickens. The detection rates of *Salmonella* spp. in feces and meat samples were 4.7% and 11.9%, respectively, which were lower than the prevalence of *Salmonella* spp. in chicken feces and meat samples obtained from commercial farms in other studies in Thailand (36.2%–62.1%) [40, 41]. The low prevalence of *Salmonella* spp. corresponded to a previous observation that native chickens are more resistant to bacterial infections and colonization than commercial chickens [42].

Regarding the prevalence of *Salmonella* spp. in fecal samples, we observed that the detection rate of *Salmonella* spp. in the current study (4.7%) was slightly higher than that in Malaysian native chickens (2.5%) [18]. However, the prevalence of *Salmonella* spp. in native chickens in Iran (27.0%) [19] and in China (39.7%) [20] differed markedly. The differences may be due to differences in sample types across studies. Cloacal swab was used in the former study, whereas a combination of liver, spleen, and large intestine was used in the latter. Moreover, different chicken breeds may influence the shedding of *Salmonella* spp. [43]. These factors might affect detection sensitivity and alter the prevalence of *Salmonella* spp. in native chickens.

### Age-related fecal shedding and seasonal influence

At the farm level, *Salmonella* spp. first appeared in fecal samples at 3 weeks of age (5.2%). The absence of *Salmonella* spp. in fecal samples from Blackbone chickens aged 1 day to 3 weeks could be due to protection from maternal antibodies [44]. Thereafter, the shedding of *Salmonella* spp. significantly increased and reached the highest level in 7-week-old chickens (20.0%) (Table 1). The increase in the detection rate of *Salmonella* spp. in Blackbone chicken feces could occur because of horizontal transmission from other colonized chicken, contaminated environment, drinking water, feeds, rodents, and insects. Moreover, in our study, the high prevalence of *Salmonella* spp. in chickens was observed at 7 weeks of age in mid-September, during the rainy season in Thailand. This result corresponded to a previous report showing a significantly higher number of *Salmonella* spp. in chicken meat during the rainy season [45].

Then, *Salmonella* spp. shedding decreased when the chickens were older, although the number of *Salmonella* spp. fluctuated between 0.8% and 10.8%. At the end of the rearing period (50 weeks), the prevalence of *Salmonella* spp. in fecal samples was 3.1% (Table 1). The decline in *Salmonella* spp. in fecal samples may have resulted from increased serum antibody production, which may reduce bacterial numbers in the intestinal tract [46]. A downward trend in the detection of *Salmonella* spp. was also observed in the meat samples (Table 1). These results were similar to those of previous studies on other poultry species, such as laying hens [47], turkeys [48], and ducks [49]. In chickens at 16 weeks of age, the prevalence of *Salmonella* spp. in meat was statistically higher than that in fecal samples (Figure 2), possibly due to the contamination of animal feces during slaughtering and processing [1].

### Distribution of serotypes and epidemiological significance

The distribution of serovars of *Salmonella* spp. varies from country to country and over time. Among the more than 2,600 *Salmonella* serovars, *S*. Enteritidis and *S*. Typhimurium are the most prevalent and widely disseminated serovars worldwide [1]. Recently, in Thailand, common serovars among food-producing animals, farms, environments, and human clinical specimens (both invasive and non-invasive diseases) were Typhimurium, Enteritidis, Rissen, Agona, Schwarzengrund, Choleraesuis, Corvallis, Weltevreden, and Altona [3, 8, 10, 13, 50].

As opposed to previous studies, our study demonstrated that *S*. Bovismorbificans was the predominant *Salmonella* serovar from Blackbone chickens, accounting for 64.1% of all isolates, regardless of sample origins and animal ages (Table 1). It is worth noting that *S*. Bovismorbificans was a predominant serovar in fecal samples until the chickens reached the age of 16 weeks; thereafter, *S*. Corvallis has replaced *S*. Bovismorbificans were reared from 22 weeks until the end of the rearing period (Table 1). The reason for this remains unknown; however, the decrease in *S*. Bovismorbificans might be associated with an increase in flock immunity to *S*. Bovismorbificans, probably due to exposure to this serovar in younger birds [51].

Although several *Salmonella* spp. serovars have been reported to be involved in foodborne outbreaks in different geographical regions, *S*. Bovismorbificans is infrequently associated with human infections. *S*. Bovismorbificans began to gain more attention in 2011 when it was implicated in a gastroenteritis outbreak in the United States [52]. Thereafter, *S*. Bovismorbificans gastroenteritis outbreaks have been reported in Europe (Netherlands, France, Germany, and Switzerland) from 2015 to 2021 and have been linked to vegetables, uncooked ham, and pork sausage [2, 53, 54]. Furthermore, a gastroenteritis outbreak in southern Greece has been associated with *S*. Bovismorbificans from tap water [4]. Moreover, *S*. Bovismorbificans is a common cause of invasive *Salmonella* spp. infections in sub-Saharan Africa [55].

In Thailand, *S*. Bovismorbificans is relatively rare and has only been detected in a limited number of samples from patients with diarrhea and food-producing animals (chickens and pigs) [8, 40, 56]. Although the role of *S*. Bovismorbificans in human gastroenteritis among Thai people remains unclear, other *Salmonella* serovars found in our study, including *S*. Corvallis, *S*. Weltevreden, *S*. Stanley, and *S*. Typhimurium, has been implicated in gastroenteritis and invasive diseases in Thai patients [3, 8, 10].

### Trends in antibiotic resistance and public health implications

The incidence of antibiotic-resistant *Salmonella* spp. isolates, including those resistant to ciprofloxacin and third-generation cephalosporins, from different origins have been increasingly documented. High rates of resistance to ampicillin (30.3%–41.0%), trimethoprim-sulfamethoxazole (4.6%–14.0%), and ciprofloxacin (8.0%–23.9%) have been observed in *Salmonella* spp. isolated from commercial poultry farms in Thailand [3, 11]. However, our study revealed that a limited number of *Salmonella* spp. isolates from Blackbone chickens were resistant to antibiotics (Figure 3 and Table 2), probably due to a minimal antibiotic exposure in a small-scale farm. These results suggest that Blackbone chickens may be used as a potential ecological model of low AMR to study the baseline and evolution of antibiotic resistance in poultry.

Compared with the *Salmonella* spp. isolates from Thai patients, the resistance rates of *Salmonella* spp. from Blackbone chickens were much lower than those from patients, such as 8.5% versus 31.3%–81.0% and 0.7% versus 25.3%–56.1% of isolates for ampicillin and trimethoprim-sulfamethoxazole, respectively (Figure 3) [3, 8, 9]. Similar observations were observed when our results were compared with those from the National AMR Surveillance Center, Thailand, in 2022 (n = 3,502), which revealed that 43.8% and 15.2% of clinical *Salmonella* spp. isolates were resistant to ampicillin and trimethoprim/sulfamethoxazole, respectively [57]. Moreover, only 1.4% of isolates from Blackbone chickens demonstrated MDR phenotypes (Table 2), whereas 52.8%–77.5% of MDR *Salmonella* spp. isolates from patients were observed [9, 10]. These data suggest that administering antibiotics is still effective for the treatment of infections caused by *Salmonella* spp. in Blackbone chickens.

Several *Salmonella* spp. isolates were placed in the intermediate category for ceftazidime (43.0%), ciprofloxacin (68.3%), and cefotaxime (71.1%) (Figure 3) with zone diameters (mean ± SD) of 19.9 ± 0.39 mm, 25.7 ± 1.62 mm, and 24.0 ± 0.7 mm, respectively. In the future, these isolates can evolve to become fully resistant. Monitoring of antibiotic-resistant *Salmonella* spp. should be performed regularly because resistance development could occur as a natural evolutionary process even in the absence of selective pressure. Horizontal transfer of antibiotic-resistant genes from other environmental bacteria to *Salmonella* spp. may also occur. This was supported by Van Meervenne *et al*. [58], who demonstrated that an environmental-resistant plasmid could be transferred to *Salmonella* spp. at high frequency.

### Genomic findings and virulence-associated characteristics

Previous studies have shown that the common *S*. Bovismorbificans strain associated with *Salmonella* spp. outbreaks in Europe and invasive infections in Africa were associated with ST142 [39–41]. However, in this study, five *S*. Bovismorbificans isolates were typed as ST1499. Although the genome pathotyping study has placed ST142 and ST1499 in the same cluster, they showed significant sequence divergence [59]. *S*. Bovismorbificans ST1499 has been reported from patients and foods in several countries [59, 60]; however, it is considered a relatively uncommon sequence type. In Thailand, *S*. Bovismorbificans ST1499 was detected in a pork sample in 2013 (Biosample: SAMN04431473); however, data related to this finding were unavailable. To the best of our knowledge, this is the first report of *S*. Bovismorbificans ST1499 was identified in poultry in Thailand, expanding the known geographic distribution of this lineage.

All isolates harbored a limited number of ARGs, consistent with the finding that most remained susceptible to antibiotics (Table 2). The presence of *bla*_TEM-1_ in *Salmonella* spp. isolates corresponded to their ampicillin-resistant phenotypes (Figure 4). Isolate CCS2 carried no *tet*, but doxycycline resistance was observed, possibly by other mechanisms, such as ribosomal protection and enzymatic inactivation [61]. In contrast, the presence of *aac* and *ant*, conferring resistance to aminoglycosides, was detected in all 6 isolates, aminoglycoside resistance was found in only two isolates. Similarly, four isolates carried *qnrS1*; however, ciprofloxacin resistance was not observed. These results suggest that *Salmonella* spp. carry silent antibiotic-resistant genes without exhibiting corresponding resistant phenotype. This observation is of great concern because resistant genes can be activated under certain conditions, leading to the development of antibiotic resistance in bacteria [62].

Plasmids are important vehicles for the conjugative transfer of genes. In this study, five isolates carried an IncI1 plasmid, a broad-host-range plasmid that typically harbors multiple ARGs. This is not surprising, as the IncI1 plasmid is frequently found in Enterobacterales of food-animal origin, including *Salmonella* spp. [63]. All the isolates that possessed the IncI1 plasmid carried *bla*_TEM-1_ (Figure 4), suggesting that *bla*_TEM-1_ may reside on this plasmid. Interestingly, two of the IncI1 plasmid-carrying isolates possessed *cib*, the gene encoding for bacteriocin (colicin Ib), consistent with the previous observation that *cib* is commonly carried on IncI1 plasmid [63]. Cib can inhibit a number of Gram-positive and Gram-negative bacteria; therefore, the *cib*-positive *Salmonella* spp. may have advantages in intestinal colonization over microbiota and readily cause infections. Co-occurrence of plasmid–borne *bla*_TEM-1_ and cib possesses an adaptive feature promoting intestinal persistence, not previously reported in Thai native chickens.

Chromosome-encoded SPI-1 and SPI-2 are necessary for *Salmonella* pathogenesis. Both islands encode T3SS-1 and T3SS-2, which are needle-like structures that can be used to inject virulence factors directly into host cells. In addition, several VF-associated genes involved in intestinal mucosa invasion are located on SPI-1, whereas those on SPI-2 are associated with bacterial survival and multiplication within host cells [64]. This study revealed that all isolates carried *prg* and *ssa*, which are required for the needle structures of T3SS-1 and T3SS-2, respectively (Figure 5). VF genes responsible for *Salmonella* invasion (*invA*) and replication within host cell (*sifA*) were found on SPI-1 and SPI-2 of all isolates. These results suggest that *Salmonella* spp. isolates from Blackbone chickens can establish infections.

Interestingly, we identified a single *S*. Weltevreden isolate, CSS26, is known as ST365, which is considered an epidemic clone worldwide. It has been found in animals, food, and environmental samples from several continents, such as Europe, America, Oceania, and Thailand [36, 48]. Moreover, *S*. Weltevreden ST365 has been implicated in gastroenteritis outbreaks in China [65]. Southeast Asia, *S*. Weltevreden ST365 is a common cause of gastroenteritis in children hospitalized in Vietnam [66] and has been detected in chicken and beef in Laos [67]. In Thailand, *S*. Weltevreden ST365 has been detected in canal water in Bangkok [50], in pigs [68], and in patients in northeastern Thailand (Biosample: SAMN16713980). This is the first report of *S*. Weltevreden ST365 isolated from native chicken in Thailand.

The CSS26 strain lacked some fimbrial adhesin genes as well as the *lpf* and *tcf* operons, which may result in the decreased colonization ability of different intestinal tissues. However, this colonization may be slightly affected because other adhesins acted simultaneously during infection [69]. Notably, the CSS26 strain is probably invasive because it carries the non-fimbrial adhesin gene *ratB*, which has been reported to be present only in invasive strains [70]. Strain CSS26 also harbored an IncFII(S) plasmid with the highest identity to pSH17G0407, which has been shown to contribute to the *S*. Weltevreden ST365 invasion capacity [65].

The chromosome-encoded ACE T6SS gene cluster, which has been reported to be associated with gut bacteria killing and outcompeting [71], is present in CSS26. Consequently, the presence of *ratB*, IncFII(S) plasmid, and ACE T6SS in CSS26 strain may play roles in intestinal cell invasion during infection. Moreover, the CSS26 strain possessed cytolethal distending toxin B (*cdtB*), a DNase that induces single- or double-strand DNA breaks in the host chromosome, leading to genetic instability. Therefore, CdtB may contribute to cancer development, although the association between CdtB and its clinical relevance remains to be elucidated [72].

Note that all 6 *Salmonella* spp. isolates carried many virulence genes but harbored only a few resistance genes. This could be considered a trade-off between antibiotic resistance and virulence, as previously observed in *Salmonella* spp. [73]. This observation could help understand the evolution of *Salmonella* spp. and develop treatment options to address antibiotic resistance.

### Phylogenetic relationships

The cgSNP analysis revealed that five *S*. Bovismorbificans ST1499 strains from Blackbone chickens were phylogenetically grouped together, possibly because they originated from the same farm (Figure 6). They were placed near the strains CFSAN045337 and GSJ/2016-Sal-017, from pork previously collected in Thailand in 2013 (Biosample: SAMN04431473) and native chicken, originating in Guangzhou, South China, in 2016 (Biosample: SAMN14309717), respectively (Figure 6). Unfortunately, data related to the sampling location of the Thai strain are not available. Therefore, the connection between our *S*. Bovismorbificans strain and CFSAN045337 remains unclear. However, our strains shared the same sequence type (ST1499) with the Thai and Chinese strains, and it is possible that *S*. Bovismorbificans from Thai Blackbone chickens, CFSAN045337 and GSJ/2016-Sal-017, share a common evolutionary history

In contrast, cgSNP analysis revealed that *S*. Weltevreden CSS26 occurred as a singleton in the phylogenetic tree (Figure 7). These results suggest that CSS26 is related to other *S*. Weltevreden strains. ST365 strains were included in the analysis, regardless of strain origin or location. These results imply that *S*. Weltevreden ST365 from Blackbone chicken may represent a unique lineage and have developed distinct evolutionary pathways.

## CONCLUSION

This longitudinal investigation provides comprehensive evidence on the occurrence, AMR, and genomic characteristics of non-typhoidal *Salmonella* in Thai native Blackbone chickens. The overall prevalence of *Salmonella* spp. was relatively low (6.3%), with detection rates of 4.7% in fecal samples and 11.9% in meat samples. Fecal shedding was age-dependent, peaking at 7 weeks of age and declining thereafter, while meat contamination was observed across all production stages. *S*. Bovismorbificans was the predominant serovar, followed by *S*. Corvallis and *S*. Weltevreden. AMR levels were generally low, with more than half of the isolates susceptible to all tested antibiotics and only 1.4% exhibiting MDR. WGS revealed that *S*. Bovismorbificans isolates belonged to ST1499 and that the *S*. Weltevreden isolate belonged to the globally distributed ST365, with all sequenced isolates harboring extensive virulence gene repertoires despite limited resistance determinants.

The detection of potentially invasive and zoonotic *Salmonella* lineages in Blackbone chickens highlights a food safety risk associated with traditional poultry production systems, particularly during slaughter and processing. The low prevalence of AMR suggests that small-scale native chicken farms with minimal antimicrobial use may serve as important reservoirs of *Salmonella* with preserved antimicrobial susceptibility. These findings underscore the importance of targeted hygiene interventions at critical points of production and slaughter, as well as continued surveillance to prevent transmission to humans within a One Health framework.

A major strength of this study lies in its prospective longitudinal design, which enabled age-specific assessment of *Salmonella* shedding and meat contamination throughout the rearing cycle. The integration of phenotypic antimicrobial susceptibility testing with WGS provided robust insights into resistance mechanisms, virulence potential, and phylogenetic relationships. Moreover, this study represents the first report of *S*. Bovismorbificans ST1499 and *S*. Weltevreden ST365 in Thai native Blackbone chickens, contributing novel baseline data to national and regional surveillance efforts.

Several limitations should be acknowledged. The study was conducted on a single farm, which may limit generalizability to other native poultry systems. Environmental samples, including water, feed, litter, and slaughterhouse surfaces, were not collected, restricting the ability to identify transmission pathways. In addition, WGS was performed on a limited number of isolates, which may underestimate the genetic diversity of circulating *Salmonella* strains.

Future studies should incorporate multi-farm sampling across different geographic regions and include comprehensive environmental and slaughterhouse surveillance to better elucidate transmission dynamics. Expanded WGS of larger numbers of isolates is warranted to monitor the emergence of resistance, virulence evolution, and interspecies transmission. Longitudinal One Health studies linking poultry, human, and environmental isolates would further strengthen risk assessment and inform evidence-based control strategies.

In conclusion, Thai native Blackbone chickens harbor non-typhoidal *Salmonella* with low AMR but substantial virulence potential. While current antimicrobial efficacy appears preserved, the presence of globally relevant and potentially invasive lineages emphasizes the need for sustained surveillance, improved biosecurity, and food safety interventions to mitigate zoonotic risk and safeguard public health.

## DATA AVAILABILITY

The associated metadata (e.g., serovar, resistance phenotype, and sample source) are included in this study. The raw sequences of *Salmonella* spp. isolates from this study have been submitted to the National Center for Biotechnology Information under the BioProject number PRJNA1147903. The assembled genomes and metadata for strains CCS2, CSS10, CSS26, CSS48, MLSS61, and MLSS62 are publicly available under BioSample accession numbers SAMN43174013, SAMN43174014, SAMN43174015, SAMN43174016, SAMN43174025, and SAMN43174026, respectively.

## AUTHORS’ CONTRIBUTIONS

KA: Methodology, fieldwork, performing experiments, data collection, curation, and analysis. AK and UT: Methodology and performing experiments. PRN: Fieldwork, data analysis and interpretation, and drafting the manuscript. PRN and TL: Conceptualization, supervision, methodology, data analysis and interpretation, and reviewing and editing the manuscript. All authors have read and approved the final version of the manuscript.

## COMPETING INTERESTS

The authors declare that they have no competing interests.

## PUBLISHER’S NOTE

Veterinary World remains neutral with regard to jurisdictional claims in the published institutional affiliations.
